# An increase in Semaphorin 3A biases the axonal direction and induces an aberrant dendritic arborization in an in vitro model of human neural progenitor differentiation

**DOI:** 10.1186/s13578-022-00916-1

**Published:** 2022-11-08

**Authors:** Gabriella Ferretti, Alessia Romano, Rossana Sirabella, Sara Serafini, Thorsten Jürgen Maier, Carmela Matrone

**Affiliations:** 1 Unit of Pharmacology, Department of Neuroscience, School of Medicine, University of Naples “Federico II”, Naples, Italy; 2 CEINGE-Advanced Biotechnologies Research Center S.C.a.R.L, University of Naples “Federico II”, Naples, Italy; 3 Paul-Ehrlich Institute, Federal Institute for Vaccines and Biomedicines, Langen, Germany

**Keywords:** Semaphorin 3A, Neurodevelopment, Neuroinflammation, Microglia, Neural progenitors

## Abstract

**Background:**

Semaphorins (Sema) belong to a large family of repellent guidance cues instrumental in guiding axons during development. In particular, Class 3 Sema (Sema 3) is among the best characterized Sema family members and the only produced as secreted proteins in mammals, thereby exerting both autocrine and paracrine functions. Intriguingly, an increasing number of studies supports the crucial role of the Sema 3A in hippocampal and cortical neurodevelopment. This means that alterations in Sema 3A signaling might compromise hippocampal and cortical circuits and predispose to disorders such as autism and schizophrenia. Consistently, increased Sema 3A levels have been detected in brain of patients with schizophrenia and many polymorphisms in Sema 3A or in the Sema 3A receptors, Neuropilins (Npn 1 and 2) and Plexin As (Plxn As), have been associated to autism.

**Results:**

Here we present data indicating that when overexpressed, Sema 3A causes human neural progenitors (NP) axonal retraction and an aberrant dendritic arborization. Similarly, Sema 3A, when overexpressed in human microglia, triggers proinflammatory processes that are highly detrimental to themselves as well as NP. Indeed, NP incubated in microglia overexpressing Sema 3A media retract axons within an hour and then start suffering and finally die. Sema 3A mediated retraction appears to be related to its binding to Npn 1 and Plxn A2 receptors, thus activating the downstream Fyn tyrosine kinase pathway that promotes the threonine-serine kinase cyclin-dependent kinase 5, CDK5, phosphorylation at the Tyr15 residue and the CDK5 processing to generate the active fragment p35.

**Conclusions:**

All together this study identifies Sema 3A as a critical regulator of human NP differentiation. This may imply that an insult due to Sema 3A overexpression during the early phases of neuronal development might compromise neuronal organization and connectivity and make neurons perhaps more vulnerable to other insults across their lifespan.

**Supplementary Information:**

The online version contains supplementary material available at 10.1186/s13578-022-00916-1.

## Introduction

During neuronal development, axons navigate to their targets by sensing attractive and repulsive signals through receptors located on their growth cones [1]. In particular, semaphorins (Sema) belong to a large family of guidance cues proteins, consisting of secreted (Sema 2, Sema 3, and Sema V), membrane spanning (Sema 1, Sema 4, Sema 5, and Sema 6) or glycosyl phosphatidyl inositol anchored (Sema 7A) proteins [2, 3].

An increasing number of studies have indicated a role of Sema 3A in the regulation of neurodevelopment. As such, Sema 3A has been reported to either trigger or inhibit axon repulsion in cortex [4, 5], as well as to prevent the pruning of hippocampal axons [6] or promote branching by cerebellar basket cell axons onto Purkinje cells in the cerebellar cortex [7]. Inconsistencies have been also reported in Sema 3A knock-out (KO) mice, where the lack in Sema 3A can cause either a reduction or an increase in terminal basket cell axonal arborization [8].

Both neuronal and non-neuronal cells in brain express Sema 3A, including microglia cells, astrocytes, endothelial cells and oligodendrocytes. Sema 3A expression is high during the early stages of embryonic development, but it reaches a peak only around the first postnatal week [9, 10]. Later, after birth, Sema 3A levels decline, although in adulthood its expression still persists in brain areas that retain plasticity and/or neurogenesis, such as the olfactory bulb, hippocampus, and cerebellum [11–13]. The reason why Sema 3A levels change during development and after birth, is currently unknown. However, it is clear that this pattern of expression is essential for brain development and to maintain neurons healthy in adulthood. In fact, alterations in the Sema 3A levels are detectable in the brain of patients affected by nervous system pathologies. Consistently, Sema 3A increase in the cells surrounding the ischemic area upon stroke insult in animal models of brain ischemia [14, 15]. Similarly, high levels of Sema 3A have been reported in brain from patients with multiple sclerosis [16] or in the Schwan cells from patients with amyotrophic lateral sclerosis [17] and in the hippocampus of patients with Alzheimer’s disease [18]. In addition, Sema3A levels are increased in the cerebellum and prefrontal cortex of schizophrenic or autistic subjects [19, 20] where several polymorphisms in Sema 3A or in the Sema 3A receptors have been also described [15, 21–27]. On the contrary, a transient downregulation of Sema3A mRNA expression has been found in a rat model of temporal lobe epilepsy [27–29]. Interestingly, increased levels of Sema 3A have been also detected in autoimmune diseases, supporting the role of Sema 3A as modulator of the inflammatory response [30, 31].

All together these observations have given rise to speculations whether an increase in Sema 3A during the early stages of neuronal development may result in alterations in growth and differentiation that can compromise neuronal functions in adulthood.

The effects of Sema 3A depend on the binding to its receptors, the neuropilin (Npn) and plexin (Plxn) A protein families [32]. This binding results in dynamic changes in the cytoskeleton and repulsive mechanisms at growth cone level [33]. In particular, Neuropilins (Npn 1 and 2) have been described to be essential for the Sema 3A binding to Plexin (Plxn) A receptors and for the activation of Sema 3A downstream signals [34]. Remarkable, Sema 3A appears to prioritize the binding to Npn 1 rather than Npn 2, in order to activate axonal repellent downstream signals [35, 36].

The goal of this study is to elucidate how neurons sense and respond to exogenous or endogenous changes in Sema 3A expression levels during the first days of development using human neural progenitors (NP). Our findings highlight a novel mechanism in which an increase in Sema 3A levels activates axonal targeting errors and branch pruning defects in NP during the first days of differentiation. These alterations might likely recapitulate possible pathological conditions that compromise neuronal functions during the early stages of development, thus affecting the neuronal growth and differentiation and predisposing to other neurologic diseases.

## Materials and methods

### Human neural progenitor cultures

Human neural progenitors (NP) (#ax0015) were obtained from Axol Bioscience (Cambridge, UK) and cultured following the procedures previously reported [37, 38]. According to the customer suggestions, NP were passaged maximum three times before using for the experiments. Shortly, NP were plated in precoated wells as well as slides using Geltrex coating solution (ThermoFisher, Milan, IT), and differentiated in Neurobasal supplemented with B27 media (ThermoFisher, Milan, IT). As controls for our experiments, some key results were confirmed also in #ax0016 (Axol Bioscience, Cambridge, UK).

For RNA silencing, 2 days after plating, NP (2,500,000 cell/well, diameter/well 35 mm) were incubated with a siRNA mix containing Sema 3A (Ambion, Milan, IT, #S20284) or Npn 1 (Ambion, Milan, IT, #107,267) or Plxn A2 siRNA (5 pmol, Ambion, Milan, IT, #S10700), 1 µl Lipofectamine Messenger Max mRNA Transfection Reagent (Invitrogen, Milan, IT) and 100 µl Opti-MEM medium (ThermoFisher, Milan, IT) and left in Neurobasal supplemented with B27 media (ThermoFisher, Milan, IT) for additional 48 h. In preliminary experiments to set working conditions, we used 5 pmol Silencer GAPDH siRNA (Ambion, Milan, IT, #AM4624) as positive control and Silencer Negative Control siRNA (Ambion, Milan, IT, #AM4611) as the negative control following supplier’s suggestions (data not shown).

For DNA transfection, 10 µg/ml of Sema 3A-GFP (OriGene Technologies Inc., Rockville, MD, USA, #RG213681) or GFP empty vector (OriGene Technologies Inc., Rockville, MD, USA #PS100010) were incubated in 1 µl Lipofectamine Stem Transfection Reagent (Invitrogen, Milan, IT) for 20 min and then the mix was transferred to NP that were left in Neurobasal supplemented with B27 media for 48 h. After 48 h, the medium was refreshed, and cells were cultured for additional 24 h.

In siRNA and DNA co-transfection experiments, NP were incubated with 10 µg/ml Sema 3A-GFP or GFP-empty vector and 5 pmol siNpn 1 (Ambion, Milan, IT, #107,267), siSema 3A (Ambion, Milan, IT, #S20284) or siPlxn A2 (Ambion, Milan, IT #S10700) in 1 µl Lipofectamine Stem Transfection Reagent (Invitrogen, Milan, IT) and left in Neurobasal supplemented with B27 media for 48 h.

### Human primary microglia

Human fetal brain-derived primary cultures of microglia (HMC3, 37,089–01), purchased from Celprogen Inc. (Benelux, NL), were cultured in Essential medium 8 (ThermoFisher, Milan, IT), that is routinely used for stem cells grow and expansion and we previously used to keep in culture NP [37, 39]. DNA transfection was performed by incubating 10 µg/ml of Sema 3A with 15 µl of Lipofectamine 2000 (Invitrogen, ThermoFisher, Milan, IT), according to the manufacturer protocols. After 20 min, fresh media was added, and cells were left in culture for 48 h. 10 µg/ml of GFP-empty vector was used as transfection positive control.

Media from Sema 3A, GFP or non-transfected microglia was collected 48 h after transfection and transferred to NP culture. In order to minimize events related to changes in growth conditions, NP were cultured in Essential medium 8 (ThermoFisher, Milan, IT) at least 48 h before the experiment.

### Western blot analysis (WB)

For protein isolation, cells were collected and homogenized in RIPA buffer (ThermoFisher, Milan, IT) supplemented with protease inhibitors (Sigma-Aldrich, Darmstadt, DE). After 60 min incubation on ice, the homogenates were centrifuged (14,000 rpm, 4 °C, 20 min) and soluble protein samples were stored at -80 °C until use. Protein concentration was determined with the Bradford assay. Equal amounts (30 μg) of proteins were separated on 4–15% precast polyacrylamide gel (Biorad Laboratoires, Milan, IT) under reducing conditions, transferred into PVDF membranes (Abcam, Cambridge, UK). Membranes were blocked with 5% Bovine Serum Albumin (BSA, Sigma-Aldrich, Milan, IT) in Tris-Buffered Saline-Tween (TBS-T, Biorad Laboratoires, Milan, IT) and incubated overnight with the appropriate primary antibody. Anti-mouse or anti-rabbit secondary antibodies (Santa Cruz Biotechnology, Dallas, TX, USA) were used to detect the primary antibody. The detection of the protein of interest is achieved using chemiluminescent method utilizing Clarity Western ECL Substrate (Biorad Laboratoires, Milan, IT). For digital quantification, densitometric analysis of the immunoreactive bands was performed using ImageLab 6.1.0 software (2020, Bio-Rad Laboratories, Milan, IT). The following primary antibodies were used (see also key resource Table): anti-β-actin (1:20,000, Sigma-Aldrich, #A3854), anti-iNOS (1:1000, Proteintech, #18,985–1-AP), anti-TNFα (1:1000, Proteintech #17,590–1-AP), anti-Sema 3A (1:1000, Invitrogen, #PA5-67,972), anti-Fyn (1:1000, Cell Signaling, #4023), anti-pFyn Tyr420 (1:1000, Cell Signaling, #2101S), anti-p35/CDK5 (1:1000, Cell signaling, #2506), anti-pCDK5 Tyr15 (1:1000, Cell Signaling, #94,254). The following secondary antibodies were used for immunoblotting: anti-rabbit IgG-HRP conjugated (1:5000, Santa Cruz, #sc-2357), anti-mouse mIgGk BP-HRP (1:5000, Santa Cruz, #sc-516102).

### Enzyme-linked immunosorbent assay (ELISA)

Sema 3A protein levels in media from microglia overexpressing Sema 3A (Sema 3A media) or GFP (GFP media) or non-transfected (Ctrl media) were assessed using Elisa kit (ELISA, Cusabio, Houston, TX, USA #CSB-E15913h), according to the manufacturer instructions. Kit sensitivity was 0,156 ng/ml, according to the manufacturer information.

### Immunofluorescence (IF)

**Fig. 1:**
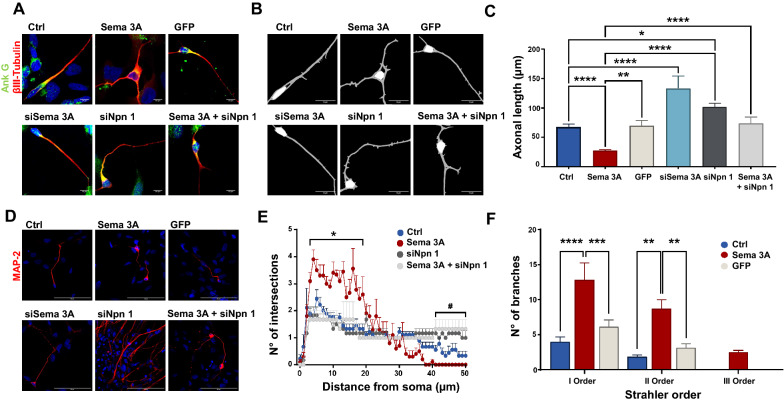
Sema 3A overexpression induces axonal retraction and increased dendritic arborization in neurons after 4 days in culture. **A** IF analysis with Ank G (green) and β-III Tubulin (red) markers of neurons transfected with: Sema 3A-GFP (Sema 3A); Sema 3A siRNA (siSema 3A); Npn 1 siRNA (siNpn 1); and Sema 3A-GFP + siNpn 1 (Sema 3A + siNpn 1). As controls, we used neurons transfected with GFP empty vector (GFP) or non-transfected (Ctrl). Scale bar: 10 µm. 40 × objective. Skeleton mask is reported in **B**. **C** Axonal length was assessed by Neuron J plug-in. Data are the mean ± SEM of three independent experiments in triplicate (approximately 100 neurons). One-way ANOVA followed by Tukey’s test for multiple comparisons. *P < 0.05; **P < 0.01; ****P < 0.0001. **D** IF analysis with MAP-2 marker (red). Scale bar: 50 µm. 40 × objective. **E** Sholl analysis was performed by Neuroanatomy plug-in of ImageJ software on MAP-2-stained neurons. Data represent the number of dendritic branches at a given distance from the soma (1 µm-radius interval) and are the mean ± SEM of three independent experiments in triplicate. Two-way ANOVA followed by Tukey’s test for multiple comparisons. *P < 0.05 vs Ctrl and.^#^P < 0.05 vs Sema 3A + siNpn 1. See also Additional File 1: Table S2 for single value analysis. **F** Dendritic branches were clustered according to Strahler orders’ classification using Neuroanatomy plug-in of ImageJ software. At least 10 neurons were analysed in each slide. Experiments were replicated three times in triplicates. Two-way ANOVA followed by Tukey’s test for multiple comparison. **P < 0.01; ***P < 0.001; ****P < 0.0001

NP were plated on precoated slides for approximately 2 days in Neurobasal supplemented with B27 media (250,000 cells/well; diameter/well 16 mm) and then transfected with siRNA or DNA or exposed to microglia conditioned media as described in each figure. NP were fixed in 4% PFA-methanol free solution (ThermoFisher, Milan, IT), washed with Dulbecco's Phosphate Buffered Saline (DPBS, ThermoFisher, Milan, IT) and permeabilized with 0.05% Triton X-100 (Bio-Rad Laboratories, Milan, IT) for 3–5 min at room temperature. Triton was eliminated and cells were rinsed with DPBS. Then, cells were processed for the staining and incubated with primary antibodies overnight at 4 °C. The primary antibodies used were: anti-Ankirin G (1:200, Invitrogen, #33–8800), anti-βIII Tubulin (1:1000, Abcam, #ab Ab18207), anti-CD68 (1:1000, Proteintech, #66,231–2-Ig) anti-CD86 (1:1000, Proteintech, #13,395–1-AP), anti-IBA1 (1:1000, Proteintech, #66,827–1-Ig), anti-iNOS (1:1000, Proteintech, #18,985–1-AP), anti-MAP-2 (1:1000, Invitrogen, #PA517646) anti-Npn 1 (1:1000, Abcam, #ab81321), anti-Plexin A2 (1:1000, Cell Signaling, #5658), anti-TMEM119 (1:1000, Proteintech, #66,948–1-Ig) anti-TNFα (1:1000, Cell Signaling, #3707). Fluorescent secondary antibodies conjugated to Alexa 488 (1:250, Invitrogen, #A-11029) or 594 (1:250, Invitrogen, #R37117) were used for primary antibodies’ detection for 45 min at room temperature. Nuclei staining was obtained with DAPI (Fluoroshield Mounting Medium with DAPI, Abcam, #AB104139) and imaged with Plan Apochromat 40x/1,3 Oil DIC M27 (Zeiss, Oberkochen, Germany) or EC Plan Neofluar 20x/0,50 M27 (Zeiss, Oberkochen, Germany) or EC Plan Neofluar 10x/0,30 (Zeiss, Oberkochen, Germany) objectives on a Zeiss LSM700 AxioObserver laser scanning confocal microscope equipped with a gallium arsenide phosphide photomultiplier tube (GaAsp-PMT) detector and controlled by a Zen black software (Zeiss, Oberkochen, Germany). Fluorescence images presented are representative of cells imaged in at least three independent experiments and were processed with Fiji, ImageJ2 software (National Institutes of Health, Bethesda, Marlan, USA). In order to analyze axonal length and dendrite organization, NP were plated onto glass slides coated in a 24-well plate at a density of 75,000 cells per well. Axonal length was assessed using NeuronJ plug-in (ImageJ); Sholl analysis and Strahler analysis were performed using Neuroanatomy plug-in (ImageJ). Representative skeleton masks in Figs. 1B and 3A were obtained using Synapse and Neurites Detector (SynD) software [40].

**Fig. 2:**
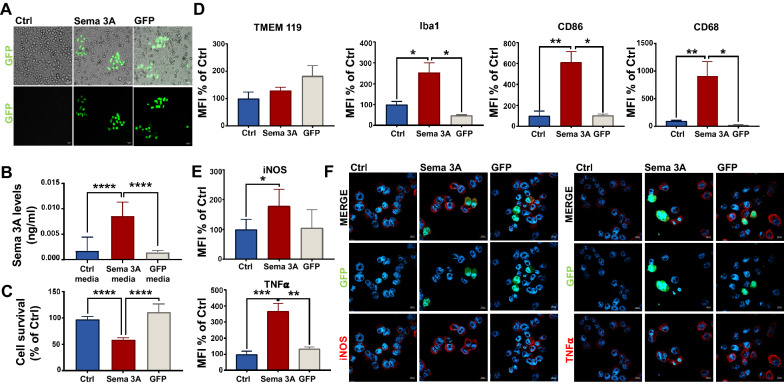
Sema 3A activates the microglia proinflammatory M1 phenotype 48 h after transfection. **A** Representative immunofluorescence pictures of the percentage of microglia cells transfected with Sema 3A-GFP (Sema 3A) or GFP empty vector (GFP). As control, we used non-transfected microglia cells (Ctrl). Scale bar: 55 µm. 10 × objective. **B** ELISA assay on media from Sema 3A, GFP and Ctrl microglia 48 h after transfection. Sema 3A levels were expressed as ng/ml and normalized on the number of alive cells (mean ± SEM from 10 different fields) for each experimental point. Data are the mean ± SEM of three independent experiments performed in triplicate. ****P < 0.0001 vs Sema 3A media. **C** Extent of cell survival obtained by counting the number of DAPI positive nuclei before and after Sema 3A transfection as well as in GFP and Ctrl microglia. Data are the mean ± SEM of three independent experiments in quadruplicate. One-way ANOVA followed by Tukey’s test for multiple comparisons. ****P < 0.0001 vs Sema 3A. **D** Quantitative analysis of TMEM 119, Iba1, CD86, CD68, and **E** iNOS and TNFα positive cells. MFI was performed on the entire slide using Zeiss Celldiscoverer7. Values are normalized on the number of DAPI positive cells for each slide and expressed as % of Ctrl. Data are the mean ± SEM of three independent experiments performed in quadruplicate. One-way ANOVA followed by Tukey’s test for multiple comparisons. *P < 0.05; **P < 0.01; ***P < 0.001. Representative images of iNOS (red) and TNFα (red) and Sema 3A or GFP (green) staining are reported in **F**. Scale bar: 10 µm. 63 × objective

Pictures in Fig. 2F were acquired with the LMS980 confocal microscope (Zeiss, Oberkochen, Germany) equipped with a Plan Apochromat 63x/1,40 Oil DIC M27 objective (Zeiss, Oberkochen, Germany), using a GaAsP-PMT detector (Zeiss, Oberkochen, Germany) and a Zen Blue Software (Zeiss, Oberkochen, Germany). Quantitative data of microglia were obtained after acquisition of huge tile regions (at least 64 frames) with Celldiscoverer7 system (Zeiss, Oberkochen, Germany) and measured Fluorescence Mean Intensity (MFI) through analysis module of Zen 3.1 Software (Zeiss, Oberkochen, Germany) (Fig. 2E). For each marker and each experiment, a threshold for the MFI was established and all the cells with higher level of fluorescence were counted as positive for that specific marker. Percentage of positive cells was calculated on total number of nuclei in the field.

### Live imaging

Live Imaging analysis was performed at CEINGE Advanced Light Microscopy Facility using the automated platform Celldiscoverer7 system (Zeiss, Oberkochen, Germany) equipped with a heated stage (37 °C and 5% CO_2_) and an Orca flash 4.0 camera (Hamamatsu). Briefly, timelapse of 6 well plates containing NP exposed to Sema 3A, GFP and Ctrl media were acquired using a Plan Apochromat 20x/0.7 objective and 1 × tubelens. Images (24 frames) were captured at 5 min intervals (Zeiss, Oberkochen, Germany) in phase gradient contrast. Axonal retraction was quantified by manual measuring of the distance between the neuronal soma and the axon edge, at time 0 and after 60 min of exposure to Sema 3A or control media, using the Zen 3.1 Software (Zeiss, Oberkochen, Germany).

All materials used for cell culture, WB, IF and live imaging experiments are reported in additional files (see Additional File 1: Table S1).

### Statistical analysis

Data are expressed as mean ± SEM. All of the experiments were performed at least three times. The appropriate statistical test was selected using GraphPad Prism software version 9.0 for Windows (GraphPad Software, San Diego, CA, USA) and reported in the legend for each figure.

### Data and code availability

Any additional information about this paper is available from the lead contact upon request**.**

## Results

### Sema 3A overexpression reduces the number of healthy neurons 48 h after transfection

We firstly analysed how human neural progenitors (NP) sense intracellular increase of Sema 3A expression during the first days of differentiation. Our previous studies indicate that NP start polarizing and generate axon growth cone approximately 48 h after plating [37, 38]. Then, 4–5 days later, a large portion of NP becomes microtubule-associated protein 2 (MAP-2) positive (neurons) and this portion further increases within the next 7–10 days [37–39]. Therefore, we will here refer to NP or neurons depending on whether cells have been cultivated two or four days, respectively. We firstly evaluated whether NP express Sema 3A. We found that all the NP expressed Sema 3A and its receptor Npn 1 two (data not shown) and four days after plating (Additional File 2: Fig.S1A), likely suggesting that there are not specific neuronal subtypes that prioritize Sema 3A expression and is more susceptible to Sema 3A signal, at this age. Interestingly, Sema 3A appeared largely distributed both in the cytosol and along the neurites, and largely colocalize with Npn 1 (Additional File 2: Fig.S1A). Next, to mimic an increase in Sema 3A, that may recapitulate neurotoxic events like those described by others in animal model of neuronal diseases [7, 16, 27, 41], NP were transfected with GFP-tagged Sema 3A (Sema 3A) or GFP-empty vector (GFP) 48 h after plating. As additional controls, we used non-transfected (Ctrl) and silenced Sema 3A (siSema 3A) NP. 48 h after transfection (four days in culture), neurons were collected, and cell extracts were used for western blot (WB) or immunofluorescence (IF) analysis. Quantification of WB bands indicated a 50% increase in Sema 3A expression in transfected neurons (Additional File 2: Fig.S1B). In particular, two bands were detectable approximately at 95 kDa and 130 kDa corresponding to the native Sema 3A [12, 42] and the transfected GFP tagged Sema 3A, respectively (Additional File 2: Fig.S1B). Of note, IF analysis showed a remarkable increase of Sema 3A staining (green) in the apical dendrites in neurons 48 h after Sema 3A transfection compared to non-transfected control (Additional File 2: Fig.S1A). Conversely, approximately 60% reduction in Sema 3A expression was observed in siSema 3A treated neurons (Additional File 2: Fig.S1B).

We next investigated whether Sema 3A was secreted in our experimental condition, by performing WB analysis in media from Sema 3A and Ctrl neurons. We observed a slight but significant increase in Sema 3A levels in the media from Sema 3A neurons when compared to the non-transfected controls (Additional File 2: Fig.S1C).

In addition, we noted that the number of DAPI positive cells was significantly reduced in Sema 3A neurons 48 h after transfection (Additional File 2: Fig.S1E), suggesting that Sema 3A overexpression may influence neuronal survival. Relevantly, only the 15% (± 3.2) of the total DAPI positive nuclei were properly transfected with Sema 3A, suggesting that such neurotoxicity is caused by both the secreted as well as the intracellular Sema 3A.

To evaluate whether the Sema 3A transfection activated a downstream signaling, we performed WB for Fyn tyrosine kinase. In this regard, it is worth noting that Fyn TK is a downstream effector of Sema 3A signaling, responsible for the regulation of axonal retraction and dendrite development [43, 44]. We found an evident increase in Fyn activation, assessed as increased phosphorylation of Tyr420 residue, 48 h after Sema 3A transfection, indicating that the Sema 3A signal was activated in neurons overexpressing Sema 3A (Additional File 2: Fig.S1D).

We finally examined whether Npn 1 silencing (siNpn 1) was able to protect against the Sema 3A mediated neuronal death in Sema 3A + siNpn 1 co-transfected neurons. siNpn 1 was confirmed by Npn 1 staining quantification (Additional File 2: Fig. S1F). Notably, the Npn 1 silencing in Sema 3A overexpressing neurons rescued neuronal survival to a level comparable to that of Ctrl non-transfected neurons (Additional File 2: Fig.S1E).

### Sema 3A overexpression causes axonal retraction and increases dendritic branching in neural progenitors

To visualize neuronal structure and dendritic arborization, 48 h after Sema 3A transfection, neurons were stained with Ankyrin G (Ank G, axonal initial segment, in green) [45] and β-III tubulin (neurites, in red) (Fig. 1A) or MAP-2 (dendrites, in red) (Fig. 1D), respectively, and examined by confocal microscopy. Axonal length and dendritic branching were analysed by Image J software. Skeleton mask analysis is reported in Fig. 1B. Neurons overexpressing Sema 3A showed a remarkable reduction in the axonal length when compared to Ctrl or GFP neurons (Fig. 1C). This reduction was associated with an increased dendrites’ branching, assessed by Sholl analysis (Fig. 1E). Interestingly, in Sema 3A overexpressing neurons, dendrite branches significantly increased in the areas around the soma (4–19 μm from the soma) (Fig. 1E, and Additional File 1: Table S2), suggesting that Sema 3A overexpression favours the formation of multiple branches rather than driving the formation of one mature axon. Moreover, we clustered the dendritic branches in I, II and III orders, according to Strahler criteria analysis and we found a significant increase in the branches’ number of order I and II in Sema 3A overexpressing neurons compared to both Ctrl and GFP (Fig. 1F), further underlining the role of Sema 3A in contributing to the dendritic architecture and organization.

Axonal retraction observed in neurons overexpressing Sema 3A was significantly preserved when cells were co-transfected with siNpn 1 (Fig. 1A–C). This finding suggests that Sema 3A requires Npn 1 receptor to explicate these functions in this experimental paradigm. In addition, an unstructured dendrite architecture in the distal areas from the soma (40–50 μm from the soma) was observed in Sema 3A + siNpn 1 neurons. This dendrite organization was different to that observed in Sema 3A where, instead, we found that dendrite arborization was increased in the proximal areas (Fig. 1E; see also Additional File 1: Table S2 for single value comparative Sholl analysis). Of interest, we noted that a large number of siNpn 1 neurons were positive to MAP-2 staining (Fig. 1D and Additional File 2: Fig. S1G) and showed long axons (Fig. 1C) and irregular dendrite arborization (Fig. 1E), when compared to non-transfected, Sema 3A and Sema 3A + siNpn 1 transfected. Intriguingly, such increased number in MAP-2 positive neurons was only observed in siNpn 1 but not in Sema 3A + siNpn 1 neurons where indeed the number of MAP-2 neurons was approximately the same than that assessed in Sema 3A overexpressing neurons (Additional File 2: Fig. S1G). This result seems to indicate that the siNpn 1 increase in MAP-2 positive neurons is not contingent to Sema 3A overexpression.

### Microglia overexpressing Sema 3A release Sema 3A and activate neuroinflammatory pathways

As Sema 3A can exert either autocrine or paracrine functions, we developed a different experimental paradigm, consisting in exposing neurons to an exogenous source of Sema 3A.

Therefore, we firstly evaluated whether Sema 3A overexpression induced a switch of microglia from resting to activated states. Next, we investigated whether Sema 3A elicits M1 or M2 microglia polarization. Microglia were transfected with GFP tagged Sema 3A following the procedure reported in Materials and Methods and the number of green cells were counted by IF and expressed as % of the total cell number in each slide. We found that 17.06% ± 1.1 Sema 3A and the 17.5% ± 2.9 GFP microglia (N = 6; % of the total number of plated cells) were transfected (Fig. 2A). Additionally, a significant amount of Sema 3A was released in media from microglia overexpressing Sema 3A, as confirmed by Elisa (Fig. 2B).

Notably, Sema 3A overexpression induced a large decrease in the number of DAPI positive microglia nuclei, consistent with a role of Sema 3A in promoting cell death and activating toxic processes (Fig. 2C).

We then evaluated whether Sema 3A overexpression impacts on microglia M1 or M2 polarization 48 h after GFP or Sema 3A transfection. For this, microglia were stained with antibodies against cluster of differentiation 68 (CD68) and 86 (CD86) and Ionized calcium-binding adapter molecule 1 (Iba 1), that have been reported to be expressed mainly in the activated microglia [46]. Transmembrane protein 119 (TMEM 119) was used as specific microglia marker, instead [47]. Finally, tumour necrosis factor-α (TNF-α) and inducible nitric oxide synthase (iNOS) were used as pro-inflammatory markers to recognise M1 polarized microglia [48, 49]. We therefore assessed the mean fluorescence intensity (MFI) for each marker in Sema 3A and GFP transfected microglia and we expressed such values in respect of the total cell number and as % of Ctrl (Fig. 2D, E). As shown in Fig. 2D, the number of TMEM 119 positive cells was not significantly different when Sema 3A was compared to GFP and Ctrl microglia. Differently, Iba 1, CD86 and CD68 were all increased in microglia overexpressing Sema 3A when compared to either Ctrl or GFP, indicating that Sema 3A activates microglia (Fig. 2D). Of note, it is worth mentioning that some cells overexpressing Sema 3A (green) and expressing Iba 1, showed an ameboid shape reminding the M1 phenotype previously shown in the human activated microglia [50] (Additional File 2: Fig.S2). Relevantly, the number of iNOS and TNF-α positive cells were largely increased in Sema 3A microglia when compared to GFP and non-transfected controls suggesting that Sema 3A promotes M1 polarization (Fig. 2E, F).

### Neural progenitors retract axon 60 min after exposure to media from microglia overexpressing Sema 3A and then die.

**Fig. 3:**
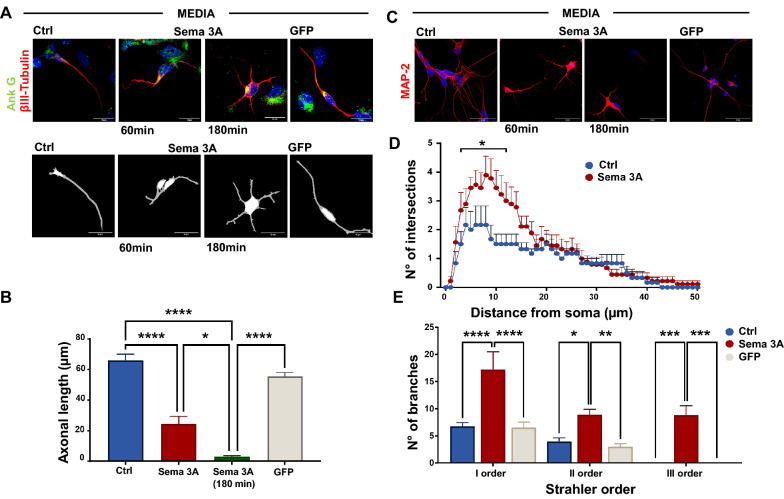
Media from microglia overexpressing Sema 3A cause axonal retraction and increase dendritic arborization in NP within an hour of exposure. **A** Representative Ank G (green) and β-III tubulin (red) staining analysis of NP after 60 min and 180 min exposure to media from microglia overexpressing Sema 3A (Sema 3A), or GFP empty vector (GFP) or non-transfected microglia (Ctrl). Scale bar: 20 µm. Figures were acquired by 40 × objective and cropped in order to visualize each single neuron. Skeleton mask is reported below. Scale bar 10 µm.The corresponding axonal length measure is reported in **B**. Analyses were performed using Neuron J plug-in. Data are the mean ± SEM of four independent experiments performed in duplicate (approximately 10 neurons for each slide). One-way ANOVA followed by Tukey’s test for multiple comparisons. *P < 0.05; ****P < 0.0001. Representative MAP-2 staining is shown in **C**. Scale bar 50 µm. **D** Dendritic branch measurements (Sholl analysis) of NP exposed to media from Ctrl, Sema 3A and GFP microglia. Data are the mean ± SEM of three independent experiments performed in duplicate (approximately 10 neurons for each slide). Analyses were performed using Neuroanatomy plug-in of ImageJ software. Two-way ANOVA followed by Tukey’s test for multiple comparisons. *P < 0.05 vs Ctrl. **E** Dendrite clustering analysis according to Strahler's order. Analyses were performed using Neuroanatomy plug-in of Image J software. Data are the mean ± SEM of three independent experiments performed in duplicate (approximately 10 neurons for each slide). Two-way ANOVA followed by Tukey’s test for multiple comparisons. *P < 0.05; **P < 0.01; ***P < 0.001: ****P < 0.0001

**Fig. 4:**
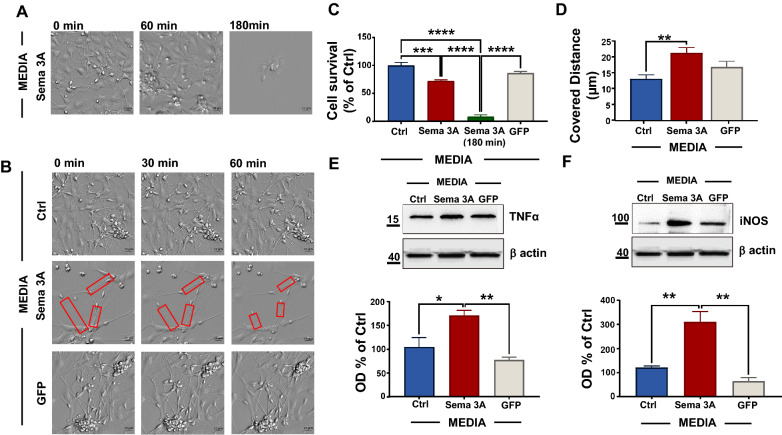
NP exposed to media from microglia overexpressing Sema 3A cover a larger distance than controls and activate a neuroinflammatory pathway. **A** Representative picture of neuronal survival 60 min and 180 min after Sema 3A media exposure. Scale bar: 20 µm. The quantitative analysis of DAPI positive nuclei is reported in **C**. Data are the mean ± SEM of three independent experiments performed in triplicate. One-way ANOVA followed by Tukey’s test for multiple comparisons. ***P < 0.001; ****P < 0.0001. **B** Representative contrast microscopy images of axonal retraction extracted from live imaging analysis (see Additional File 3, 4, 5, live imaging videos) in NP 30 and 60 min after Sema 3A, Ctrl, and GFP media exposure. Scale bar: 20 µm. **D** Covered distance of NP 60 min after Sema 3A media exposure as well as GFP or control microglia media calculated from live imaging analysis. Data are mean ± SEM of three different experiments, N = 3 (5 neurons for each experimental point in triplicate). One-way ANOVA followed by Tukey’s test for multiple comparisons. **P < 0.01. TNFα **E** and iNOS **F** WB analyses on NP after 60 min exposure to Sema 3A, GFP or Ctrl microglia media. Figure is representative of six different experiments. Optical density (OD) analysis is reported below. Data are mean ± SEM of six different experiments and are expressed as % of Ctrl. One-way ANOVA followed by Tukey’s test for multiple comparisons. *P < 0.05; **P < 0.01 vs Sema 3A

We next exposed NP to media from microglia transfected with Sema 3A (Sema 3A) or GFP (GFP) or non-transfected (Ctrl) for 60 or 180 min. 60 and 180 min after exposure, neurons were stained with Ank G (green) and β-III tubulin (red) to assess axonal length and with MAP-2 (red) for dendritic branching (Fig. 3A, C). We found that media from Sema 3A induced a significant axonal retraction (Fig. 3A, B) as also shown by skeleton mask (Fig. 3A) and an increased apical dendritic arborization (Fig. 3C, D) in NP within an hour after exposure, consistently with data reported in neurons overexpressing Sema 3A (Fig. 1). In addition, Strahler analysis showed an increase in the branches’ number of order I, II and III in neurons upon media from Sema 3A microglia (Fig. 3E), when compared to media from both Ctrl and GFP. This result indicates that an increased structural complexity occurs in the dendritic branches of NP exposed to Sema 3A when compared to NP overexpressing Sema 3A (Fig. 1F). This may suggest that NP are more sensitive to Sema 3A if exogenously administered. In addition, the number of NP exposed to media from Sema 3A microglia was largely reduced 60 min after exposure and 180 min later the majority of NP were aggregated to form big clusters in which most cells were dead (Fig. 4A, C). By contrast, NP exposed to media from GFP or Ctrl microglia neither suffered nor died (Fig. 4C).

Axonal retraction started already 30 min after Sema 3A media exposure (Fig. 4B, see also supporting materials/live imaging videos, Additional File 3, Additional File 4, Additional File 5). To quantify the axonal retraction that was shown in live imaging, we assessed the distance between the neuronal soma and the axon edge within the 60 min after exposure to media from Sema 3A microglia and we found that the distance of Sema 3A retraction approximately doubled when compared to GFP and Ctrl neurons (Fig. 4D).

Finally, NP expressed high iNOS and TNFα levels, suggesting that an exogenous Sema 3A insult from microglia M1 activated cells initiates neuroinflammatory pathways (Fig. 4E, F), that likely results in NP death (Fig. 4A, C). Of note, a similar proinflammatory pathway appeared to be also activated in microglia when exposed to media from neurons overexpressing Sema 3A. In fact, the number of iNOS (Additional File 2: Fig.S3A, C) and TNFα (Additional File 2: Fig.S3B, D) positive microglia were increased and largely died within an hour after Sema 3A neuronal media exposure (Additional File 2: Fig.S3E).

**Fig. 5:**
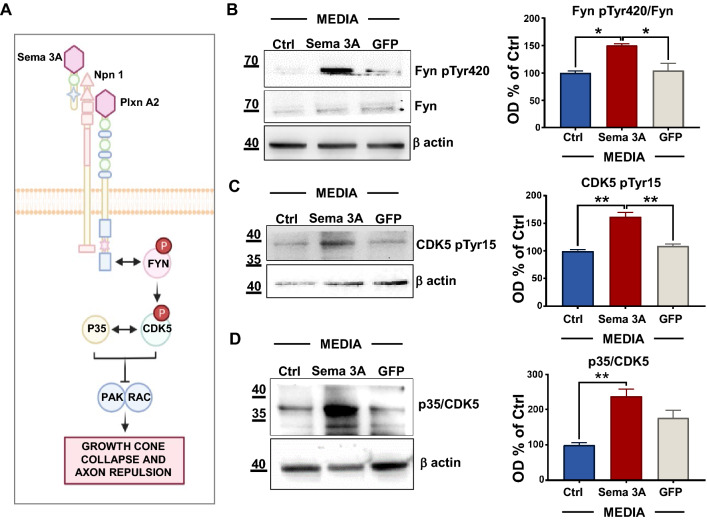
Fyn-CDK5 pathway is activated in NP 60 min after exposure to media from microglia overexpressing Sema 3A. **A** Cartoon summarizing the proposed Sema 3A downstream pathway in NP exposed to Sema 3A media for 60 min. Representative WB analyses of Fyn pTyr420 and Fyn **B**, CDK5 pTyr15 **C** and CDK5/p35 **D** as well as of the correspondent β actin. OD analysis is reported on the right. Fyn, p35/CDK5 and CDK5 pTyr15 OD values were normalized to β actin and expressed as a percentage of Ctrl. Fyn pTyr420 levels were calculated as a ratio of Fyn pTyr420 relative to the corresponding Fyn OD values (Fyn pTyr420/Fyn). N = 3. One-way ANOVA followed by Tukey’s test for multiple comparisons. *P < 0.05; **P < 0.01

We finally performed WB analysis to evaluate whether Sema 3A signal was activated, focusing on Fyn and its downstream effector, the threonine-serine kinase cyclin-dependent kinase 5, CDK5 (see also cartoon reported in Fig. 5A) [51]. Of note, we found an increase in the levels of Fyn phosphorylated at of Tyr420 (Fig. 5B) in Sema 3A neurons that did not occur in neurons upon GFP or Ctrl media, suggesting that Sema 3A activates Fyn pathway. Previously, Sasaki et al. demonstrated that Fyn phosphorylates CDK5 on the Tyr15 residue [52]. Indeed, we detected higher levels of Tyr15 CDK5 phosphorylation in neurons exposed to media from Sema 3A microglia (Fig. 5C, D). In addition, we found an increased processing of CDK5 to generate the active fragment p35 [53], consistent with a role of Sema 3A in activating the Fyn/CDK5 signaling.

**Fig. 6:**
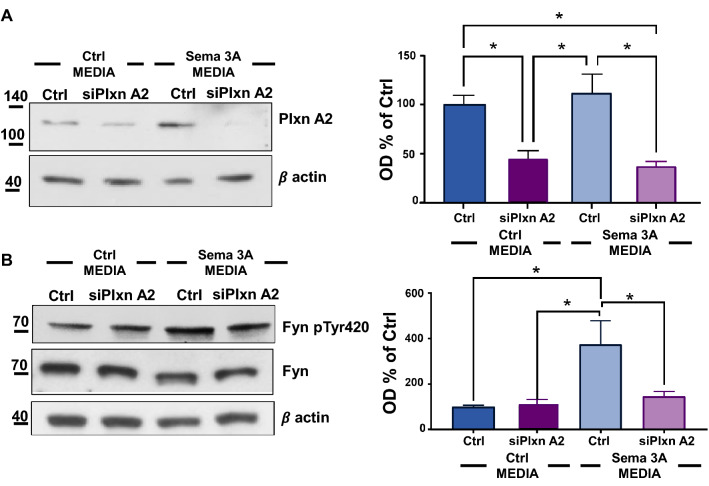
Axonal retraction and Fyn Tyr420 phosphorylation is partially prevented in siPlxn A2 neurons exposed to media from microglia overexpressing Sema 3A. Representative WB analysis of Plxn A2 **A** or Fyn pTyr420 and Fyn **B**, and of the correspondent β actin. OD analysis is reported on the right. Fyn pTyr420 levels were calculated as a ratio of Fyn pTyr420 relative to the corresponding Fyn OD values normalized to β actin (Fyn pTyr420/Fyn). N = 3. One-way ANOVA followed by Tukey’s test for multiple comparisons. *P < 0.05

As Sema 3A-Fyn-CDK5 signaling mostly involves Plxn A2 receptor activation [52], we silenced Plxn A2 (siPlxn A2) receptor expression and analysed whether neuronal exposure to media from Sema 3A microglia was still able to activate Fyn cascade. siPlxn A2 was confirmed by quantification of WB analysis (Fig. 6A). Of note, WB analysis showed reduced Fyn Tyr420 phosphorylation levels in siPlxn A2 neurons incubated in media from Sema 3A microglia (siPlxn A2 + Sema 3A MEDIA) when compared to neurons in which Plxn A2 was not silenced (Ctrl + Sema 3A MEDIA, Fig. 6B), suggesting that axonal retraction signal needs the Sema 3A binding to Plxn A2 receptor to be initiated.

## Discussion

Neurodevelopmental disorders, such as schizophrenia and autism, are chronic conditions occurring early in brain development and resulting in functional neuronal abnormalities and aberrant neuronal connectivity, mostly consisting in the abnormal sprout of neuronal processes or in altered spine growth and dendrite morphology [54–56]. Notably, recent evidences have underlined the potential critical role of Sema 3A in fostering these alterations [7, 14, 57].

An increasing number of epidemiological studies are stressing the significance of maternal inflammation in the onset of autism or schizophrenia [58–61]. In particular, prenatal exposure to infections have been described as causative factor in rise of schizophrenic births [62]. Of interest, Meyer et al. [63] demonstrated that neurons respond differently to inflammatory stimuli according to the foetal age during gestational development, delineating the hypothesis that the time of prenatal insult may differently affect neuronal structure abnormalities. This means that insults occurring at the earliest times of neurodevelopment might cause defects persisting throughout adulthood [64]. Sema 3A has been described as critical regulatory checkpoint of the immune response [65]. Sema 3A is expressed in oligodendrocytes, astroglia and microglia/macrophage where it orchestrates the innate immune response being involved in both normal and pathological immune processes [66, 67].

Multiple evidence points on microglia and neuronal interactions as crucial either in creating the specific molecular environment dictating neuronal differentiation [66–69], or triggering inflammatory pathways, impairing synaptic functions and increasing susceptibility to peripheral insults in neurodevelopmental disorders [70, 71]. Of interest, a variety of not yet defined stimuli, trigger resting microglia turning into the M1 (neurotoxic) or M2 (protective) activated phenotype [72]. In this context, Sema 3A has been reported to modulate the microglia switching from the resting to the M1 activated phenotype [73].

Of interest, Fujita et al., previously proposed a role for Sema 3A in mediating neuronal–microglia interactions after middle cerebral artery occlusion [70, 71]. Accordingly, Majed et al. demonstrated that stressed neurons can mediate death of activated microglia by increasing Sema 3A production [74]. Notably, in an animal model of spinal cord injury, Sema 3A inhibits the neuroinflammatory response against microglia [75–77]. However, much remains elusive with regard to how and whether Sema 3A may influence the neuron-microglia crosstalk during neurodevelopment.

Taking into account all these aspects, we hypothesized that increased levels of Sema 3A during the early stages of neuronal development, may result in alterations in neuronal growth or neurochemical dysregulations, such as the activation of neuroinflammatory processes. To investigate our hypothesis, we analysed how Sema 3A impacts on NP differentiation using two different paradigms consisting one in transfecting NP with Sema 3A and the other in exposing NP to media from microglia overexpressing Sema 3A. Both the paradigms affected the architecture of the NP at the very early stages of their differentiation. In particular, when Sema 3A insult came from microglia, it activated neuroinflammatory pathways in NP and induced cell death. Additionally, Sema 3A overexpression promoted the axon growth cone retraction and initiated an aberrant dendritic arborization in the area proximal to the soma, mirroring a phenotype previously described in neurons derived from patients with autism [78].

Of note, the effects on axonal elongation and branching due to Sema 3A overexpression were partially rescued in neurons in which Npn 1 and Plxn A2 were silenced, pointing on these two receptors as major transducers of Sema 3A signal in NP. While there is a large consent that Sema 3A requires Npn 1 as additional receptor moiety to create a holoreceptor complex with Plxn As and promote Sema 3A chemorepellent signalling [79], it is still controversial which of the Plxn As transduces Sema 3A signaling.

Indeed, studies report that Plxn A2 and Fyn, form a complex with Npn 1 that results in Plxn A2 phosphorylation and promotes the activation of the downstream CDK5 pathway [52, 80, 81]. In particular, Fyn, when activated, phosphorylates the Plxn A2 cytoplasmic domain [52] as well as CDK5 and forms a complex with Sema 3A receptors, Plxn A2 and Npn 1. As consequence, phosphorylated CDK5, triggers Pak/Rac signals and cytoskeletal rearrangements [52, 82].

Consistently, Sasaki et al., proposed previously a signal transduction pathway in which Sema 3A-Fyn interaction was essential in controlling apical dendrite guidance in the cerebral cortex of Sema 3A knock out mice [52]. In line with these results, we found that Fyn Tyr420 phosphorylation increased in NP exposed to Sema 3A media and that, such phosphorylation, was partially reduced in neurons in which Plxn A2 was silenced, pointing to Fyn as downstream effector of Sema 3A-Plxn A2 signaling in human neurons. Notably, although our data indicate Plxn A2 as one important player in transducing Sema 3A signal transduction, the possibility that other Plxn As might contribute to all the processes described here, cannot be ruled out.

Furthermore, future studies should elucidate which factors may induce Sema 3A overexpression in microglia as well as in neurons during neuronal development. Studies have reported increased Sema 3A expression levels in some neurologic diseases in which neuroinflammation appears to play a critical role [15, 16, 18, 83, 84]. However, as far as we know, it is still unclear which factors initiate or promote such Sema 3A increase. Of note, in these studies Sema 3A appeared to trigger either protective [31] or detrimental functions [73, 85]. We found that Sema 3A activates microglia and promotes M1 polarization. Intriguingly, iNOS and TNFα markers were also increased in neurons 60 min after exposure to Sema 3A media, indicating that a very short inflammatory insult, during the very early stages of neuronal development may promote neuroinflammatory pathways and affect differentiation and connectivity. Consistently with this hypothesis, also microglia when exposed to media from neurons overexpressing Sema 3A polarized toward the M1 phenotype and demonstrated increased iNOS and TNFα levels associated with increased cell death. These last evidences may advise about the role of other factors, beyond Sema 3A, in contributing to -or potentiating- Sema 3A effects on neuronal progenitor growth and differentiation.

All together these findings point to Sema 3A insult as potential trigger of neurodevelopmental deficits in human NP and define the downstream effectors of Sema 3A signals that are responsible for these defects. Indeed, whether such aberrant neuronal signaling plays a major role in the development of autism or schizophrenia deserves additional investigation. Furthermore, research on the mechanisms regulating the transcription, expression, and degradation of Sema 3A and leading to its accumulation in neuronal diseases should be pushed forward.

## Supplementary Information

**Additional file 1: Table S1.** Key Resource Table. **Table S2.** Dendritic branching analysis of Figure 1E. Data are the mean ± SEM of three independent experiments in triplicate. Two-way ANOVA followed by Tukey’s test for multiple comparisons. *P < 0.05; **P < 0,01; ****P < 0,0001 vs Ctrl and #P<0.05 vs Sema 3A + siNpn 1.

**Additional file 2: Fig.S1.** Characterization of Ctrl, Sema 3A-GFP, siSema 3A, siNpn 1 and Sema 3A+siNpn 1 neurons 48 h after transfection. (A) IF analysis with Sema 3A (green) and Npn 1 (red) of neurons transfected (Sema 3A) or not (Ctrl) with Sema 3A-GFP. Scale bar: 20μm. Figures were acquired by 40x objective and cropped in order to visualize single neuron and highlight the preferential Sema 3A localization on the apical dendrites in Sema 3A transfected neurons. (B) WB analysis of neurons overexpressing Sema 3A-GFP (Sema 3A) or in which Sema 3A is silenced (siSema 3A) 48 h after transfection. Non-transfected neurons were used as control. Optical density (OD) analysis is reported below. Data are the mean ± SEM of three independent experiments and are expressed as % of Ctrl. One-way ANOVA followed by Tukey’s test for multiple comparisons. *P<0.05 vs Ctrl. (C) Representative WB analysis for Sema 3A of media from NP overexpressing Sema 3A-GFP (Sema 3A) or non-transfected Ctrl. OD analysis is reported below. Sema 3A levels were normalized for the corresponding IgG value (input). One-way ANOVA followed by Tukey’s test for multiple comparisons. N=3 ****P < 0.0001. (D) Representative WB analysis of Fyn pTyr420, Fyn and the correspondent β actin. OD analysis is reported below. Fyn pTyr420 levels were calculated as a ratio of Fyn pTyr420 relative to the corresponding Fyn OD values normalized to β actin (Fyn pTyr420/Fyn). N= 3. One-way ANOVA followed by Tukey’s test for multiple comparisons. *P < 0.05. (E) Extent of neuronal survival obtained by counting the number of DAPI positive nuclei before and after Sema 3A transfection as well as in Npn 1 silencing and Ctrl (non-transfected neurons). Data are the mean ± SEM of three independent experiments in triplicate. One-way ANOVA followed by Tukey’s test for multiple comparisons. *P<0.5, ****P<0.0001. (F) Staining quantification of Npn 1 RNA silencing (siNpn 1) expressed as mean fluorescence intensity (MFI). MFI was normalized on the number of DAPI positive nuclei (three slides from three independent experiments). Scale bar: 50μm. 40x objective. Unpaired t-test, **P<0.01 vs Ctrl. (G) Analysis of the number of MAP-2 positive neurons assessed using multipoint tool of Image J. Values were normalized on the number of DAPI positive nuclei and expressed as % of Ctrl. Data are the mean ± SEM of three independent experiments in triplicate. One-way ANOVA followed by Tukey’s test for multiple comparisons. **P<0.01 vs siNpn 1. **Figure S2.** Representative images of Iba1 (red) and Sema 3A or GFP (green) staining. Scale bar: 15µm. Figures were acquired by 40x objective and cropped in order to highlight the morphology of Sema 3A or GFP transfected cells. N=3. **Figure S3.** Media from neurons overexpressing Sema 3A cause inflammatory pathway activation in microglia within an hour after exposure. iNOS (A) and TNFα (B) staining in microglia cells transfected with Sema 3A-GFP (Sema 3A) or not (Ctrl) and incubated in media from neurons overexpressing (Sema 3A neuronal media) or not (Ctrl neuronal media) Sema 3A for 60 min. Scale bar: 40 μm iNOS and 50 μm TNFα IF. 20x objective. The number of iNOS and TNFα positive cells is reported in (C) and (D), respectively. One-way ANOVA followed by Tukey’s test for multiple comparisons. N=3 *P<0.05; **P<0.01; ***P<0.001; ****P<0.0001 vs Ctrl. (E) DAPI positive nuclei measurement. Data are the mean ± SEM of five independent experiments and expressed as % of Ctrl (non-transfected microglia). One-way ANOVA followed by Tukey’s test for multiple comparisons. N=3. **P<0.01; ****P<0.0001.

**Additional file 3: **Video Ctrl. Dynamics of axonal retraction in neurons exposed to Ctrl media.

**Additional file 4: **Video Sema 3A. Dynamics of axonal retraction in neurons exposed to Sema 3A media.

**Additional file 5: **Video GFP. Dynamics of axonal retraction in neurons exposed to GFP media. NP were cultured in 6 wells plate, incubated in 50% Neurobasal supplemented with B27 and 50% E8 microglia growth media. 48 h later NP were exposed to media from microglia overexpressing Sema 3A or GFP or control for 60 min. Neuronal dynamic was recorded using Zeiss Celldiscoverer 7. Scale bar: 20μm.

## Data Availability

Data available on request from the authors.
