# Supplementary material for: An increase in Semaphorin 3A biases the axonal direction and induces an aberrant dendritic arborization in an in vitro model of human neural progenitor differentiation
Source: Cell Biosci. 2022 Nov 8;12:182. doi: 10.1186/s13578-022-00916-1 (PMC9641809; doi:10.1186/s13578-022-00916-1)
Supplement: Supplementary file 1 — Additional file 1: Table S1. Key Resource Table. Table S2. Dendritic branching analysis of Figure 1E. Data are the mean ± SEM of three independent experiments in triplicate. Two-way ANOVA followed by Tukey’s test for multiple comparisons. *P < 0.05; **P < 0,01; ****P < 0,0001 vs Ctrl and #P<0.05 vs Sema 3A + siNpn 1. [file 13578_2022_916_MOESM1_ESM.pdf]

Additional File 1: Table S1 Key Resource Table.

REAGENT or RESOURCE	SOURCE	IDENTIFIER
**Antibodies**		
**Ankyrin G Monoclonal Antibody (4G3F8)**	Invitrogen	Cat #33-8800
**Anti-bIII tubulin**	Abcam	Cat #ab195879
**Anti-bIII tubulin**	Abcam	Cat # Ab18207
**Anti-pfyn**	Cell Signaling	Cat #2101S
**Anti-β-Actin−Peroxidase antibody, Mouse monoclonal**	Sigma-Aldrich	Cat #A3854, RRID: AB_262011
**CD68 Monoclonal antibody**	Proteintech	Cat #66231-2-Ig, RRID: AB_2881622
**CD86 Polyclonal antibody**	Proteintech	Cat #13395-1-AP, RRID: AB_2074882
**CDK5 Antibody**	Cell Signaling	Cat #2506, RRID: AB_2078855
**Fyn Antibody**	Cell Signaling	Cat #4023, RRID: AB_10698604
**IBA1 Monoclonal antibody**	Proteintech	Cat #66827-1-Ig, RRID: AB_2882170
**iNOS Polyclonal antibody**	Proteintech	Cat #18985-1-AP, RRID: AB_2782960
**MAP2 Polyclonal Antibody**	Invitrogen	Cat #PA5-17646, RRID: AB_11006358
**Phospho-CDK5 (Tyr15) Antibody**	Cell Signaling	Cat #94254
**Plexin A2 (D42B5) Rabbit mAb**	Cell Signaling	Cat #5658, RRID: AB_10691688
**Recombinant Anti-Neuropilin 1 antibody [EPR3113]**	Abcam	Cat #ab81321, RRID: AB_1640739
**SEMA3A Polyclonal Antibody**	Invitrogen	Cat #PA5-67972, RRID: AB_2691930
**TMEM119 Monoclonal antibody**	Proteintech	Cat #66948-1-Ig, RRID: AB_2882272
**TNF Alpha Polyclonal antibody**	Proteintech	Cat #17590-1-AP, RRID: AB_2271853
**Goat anti-Mouse IgG (H+L) Highly Cross-Adsorbed Secondary Antibody, Alexa Fluor 488**	Invitrogen	Cat #A-11029, RRID: AB_2534088
**Goat anti-Rabbit IgG (H+L) Cross-Adsorbed ReadyProbes™ Secondary Antibody, Alexa Fluor 594**	Invitrogen	Cat #R37117, RRID: AB_2556545
**mouse anti-rabbit IgG-HRP**	Santa Cruz	Cat #sc-2357, RRID: AB_628497
**m-IgGκ BP-HRP**	Santa Cruz	Cat #sc-516102, RRID: AB_2687626
**Chemicals, peptides, and recombinant proteins**		
**10x Tris/Glycine Buffer**	Bio-Rad Laboratoires	Cat #1610734
**10x Tris/Glycine/SDS Buffer**	Bio-Rad Laboratoires	Cat #1610732
**4x Laemmli Sample Buffer**	Bio-Rad Laboratoires	Cat #1610747
**B-27™ Plus Neuronal Culture System**	ThermoFisher Scientific	Cat #A3653401
**Bio-Rad Protein Assay Dye Reagent Concentrate, 450 ml**	Bio-Rad Laboratoires	Cat #5000006
**Bovine Serum Albumin (BSA)**	Sigma-Aldrich	Cat #A3294
**Clarity Western ECL Substrate, 500 ml**	Bio-Rad Laboratoires	Cat #170-5061
**Complete™, Mini Protease Inhibitor Cocktail**	Sigma-Aldrich	Cat #11836153001
**DPBS, calcium, magnesium**	ThermoFisher Scientific	Cat #14040133
**Essential 8™ Medium**	ThermoFisher Scientific	Cat #A1517001
**Fetal Bovine Serum, heat inactivated, qualified, One Shot™, Brazil**	ThermoFisher Scientific	Cat #10108165
**Geltrex™ hESC-Qualified, Ready-To-Use, Reduced Growth Factor Basement Membrane Matrix**	ThermoFisher Scientific	Cat #A1569601
**Geneticin™ Selective Antibiotic (G418 Sulfate) (50 mg/mL**	ThermoFisher Scientific	Cat #10131035
**Image-iT™ Fixative Solution (4% formaldehyde, methanol-free)**	Invitrogen	Cat #FB002
**L-Glutamine (200 mM)**	ThermoFisher Scientific	Cat #25030024
**Lipofectamine Messenger Max mRNA Transfection Reagent**	Invitrogen	Cat #LMRNA015

**Lipofectamine™ 2000 Transfection Reagent**	Invitrogen	Cat #11668019
**Mounting Medium with DAPI - Aqueous, Fluoroshield**	Abcam	Cat #AB104139
**Opti-MEM™ I Reduced Serum Medium**	ThermoFisher Scientific	Cat #31985062
**Penicillin-Streptomycin (10,000 U/mL)**	ThermoFisher Scientific	Cat #15140122
**Ripa Buffer**	ThermoFisher Scientific	Cat #89900
**Spectra™ Multicolor Broad Range Protein Ladder**	ThermoFisher Scientific	Cat #26634
**Tris Buffered Saline 10x**	Bio-Rad Laboratoires	Cat #706435
**Triton X-100 Detergent**	Bio-Rad Laboratoires	Cat #1610407
**Trypsin-EDTA (0.05%), phenol red**	Sigma-Aldrich	Cat #T3924
**Critical commercial assays**		
**Semaphorin 3A ELISA kit**	Cusabio	Cat #CSB-E15913h
**Experimental models: Cell lines**		
**Human iPSC-Derived Neural Stem Cells (Female)**	Axol Bioscience	Cat #Ax0016
**Human iPSC-Derived Neural Stem Cells (Male)**	Axol Bioscience	Cat #Ax0015
**Human Microglia Primary Cell Culture - Frozen Vial**	Celprogen	Cat #37089-01
**Oligonucleotides**		
**Neuropilin-1 siRNA**	Ambion	Cat #AM16708 ID:107267
**PlexinA2 siRNA**	Ambion	Cat #4392420 ID: S10700
**Sema-3A siRNA**	Ambion	Cat #4392420 ID: S20284
**Silencer GAPDH siRNA Positive Control**	Ambion	Cat #AM4624
**Silencer Negative Control #1 siRNA**	Ambion	Cat #AM4611
**Recombinant DNA**		
**Semaphorin 3A (SEMA3A) (NM_006080) Human Tagged ORF Clone**	Origene	Cat #RG213681
**pCMV6-AC-GFP Mammalian Expression Vector**	Origene	Cat #PS100010
**Software and algorithms**		
**FIJI**	Schindelin et al., 2012	https://fiji.sc/; RRID: SCR_002285
**Image Lab**	Bio-Rad Laboratoires	https://www.bio-rad.com/it-it/product/image-lab-software?ID=KRE6P5E8Z
**ImageJ**	Schneider et al., 2012	https://imagej.nih.gov/ij/; RRID:SCR_003070
**Synapse and Neurite Detector (SynD)**	Schmitz, Hjorth, et al 2011	https://www.johanneshjorth.se/files/SynD/
**Prism 9**	GraphPad	https://www.graphpad.com/scientificsoftware/prism/; RRID:SCR_002798
**Zen Black**	Zeiss	RRID:SCR_018163
**Zen Blue**	Zeiss	RRID:SCR_013672
**Other**		
**12 mm Coverslips**	Epredia	Cat #CB00120RA120MZN0
**Epredia™ SuperFrost Plus™ Adhesion slides**	ThermoFisher Scientific	Cat #J1800AMNZ
**4–15% Mini-PROTEAN® TGX Stain-Free™ Protein Gels, 10 well, 30 µl**	Bio-Rad Laboratoires	Cat #4561084
**Thick Blot Filter Paper, Precut, 7.5 x 10 cm**	Bio-Rad Laboratoires	Cat #1703932
**Low Fluorescence Western Membrane (PVDF)**	Abcam	Cat #ab133411

Additional File 1: Table S2 Dendritic branching analysis of Figure 1E. Data are the mean ± SEM of three independent experiments in triplicate. Two-way ANOVA followed by Tukey's test for multiple comparisons. *P < 0.05; **P < 0,01; ****P < 0,0001 vs Ctrland #P<0.05 vs Sema 3A + siNpn 1.

µm	Ctrl vs. Sema 3A	Ctrl vs. Sema 3A + siNpn 1	Ctrl vs. siNpn 1	Sema 3A vs. Sema 3A + siNpn 1	Sema 3A vs. siNpn 1	Sema 3A + siNpn 1 vs. siNpn 1
**0**	ns (0,9857)	ns (0,9725)	ns (0,9466)	ns (0,9972)	ns (0,9943)	ns (>0,9999)
**1**	ns (0,7254)	ns (0,5604)	ns (0,6032)	ns (0,9338)	ns (0,9868)	ns (0,9899)
**2**	ns (0,8136)	ns (0,4248)	ns (0,0903)	ns (0,7917)	ns (0,3792)	ns (0,9899)
**3**	** (0,0034)	ns (0,9725)	ns (0,9991)	* (0,0237)	** (0,0077)	ns (0,9899)
**4**	**** (<0,0001)	ns (>0,9999)	ns (0,9763)	**** (<0,0001)	**** (<0,0001)	ns (0,9899)
**5**	* (0,0148)	ns (0,4248)	ns (0,4304)	** (0,0017)	*** (0,0002)	ns (0,9899)
**6**	* (0,0121)	ns (0,6976)	ns (0,5154)	** (0,0068)	*** (0,0002)	ns (>0,9999)
**7**	** (0,0013)	ns (0,9148)	ns (0,8427)	** (0,0068)	*** (0,0002)	ns (>0,9999)
**8**	** (0,0089)	ns (0,9725)	ns (0,9466)	* (0,0415)	** (0,0043)	ns (>0,9999)
**9**	** (0,0081)	ns (0,9964)	ns (0,9927)	ns (0,0697)	* (0,0102)	ns (>0,9999)
**10**	**** (<0,0001)	ns (0,9964)	ns (0,6897)	** (0,0034)	**** (<0,0001)	ns (0,9274)
**11**	**** (<0,0001)	ns (>0,9999)	ns (0,9763)	** (0,0063)	**** (<0,0001)	ns (0,9899)
**12**	** (0,0012)	ns (0,8532)	ns (0,7458)	** (0,0027)	**** (<0,0001)	ns (>0,9999)
**13**	**** (<0,0001)	ns (0,9725)	ns (0,9927)	** (0,0029)	**** (<0,0001)	ns (0,9274)
**14**	** (0,0053)	ns (0,9148)	ns (0,9763)	ns (0,3504)	** (0,0043)	ns (0,793)
**15**	** (0,0013)	ns (0,9148)	ns (0,9763)	ns (0,2053)	** (0,0012)	ns (0,793)
**16**	****(<0,0001)	ns (0,9148)	ns (0,9763)	** (0,0013)	**** (<0,0001)	ns (0,793)
**17**	** (0,0013)	ns (0,9964)	ns (0,9019)	* (0,0247)	*** (0,0004)	ns (0,9899)
**18**	* (0,0116)	ns (0,9148)	ns (0,9763)	* (0,0247)	** (0,0087)	ns (0,9899)

**19**	** (0,0019)	ns (0,9148)	ns (0,9763)	** (0,0085)	** (0,0017)	ns (0,9899)
**20**	ns (0,1133)	ns (0,9964)	ns (0,5154)	ns (0,2819)	ns (0,9353)	ns (0,6081)
**21**	ns (0,3564)	ns (0,9725)	ns (0,9991)	ns (0,3875)	ns (0,3792)	ns (0,9899)
**22**	ns 0,9502	ns (0,9964)	ns (0,9991)	ns (0,9338)	ns (0,9868)	ns (0,9899)
**23**	ns 0,1054	ns (>0,9999)	ns (0,9763)	ns (0,3875)	ns (0,3792)	ns (0,9899)
**24**	ns 0,8296	ns (>0,9999)	ns (0,9763)	ns (0,9338)	ns (0,9868)	ns (0,9899)
**25**	ns 0,9416	ns (>0,9999)	ns (0,9763)	ns (0,9789)	ns (0,9998)	ns (0,9899)
**26**	ns (0,9944)	ns (0,9964)	ns (0,9991)	ns (0,9789)	ns (0,9998)	ns (0,9899)
**27**	ns (0,9416)	ns (>0,9999)	ns (>0,9999)	ns (0,9789)	ns (0,9579)	ns (>0,9999)
**28**	ns (0,9920)	ns (>0,9999)	ns (>0,9999)	ns (0,9972)	ns (0,9943)	ns (>0,9999)
**29**	ns (0,6676)	ns (>0,9999)	ns (>0,9999)	ns (0,8579)	ns (0,7435)	ns (>0,9999)
**30**	ns (0,4485)	ns (0,9725)	ns (0,9466)	ns (0,9338)	ns (0,8734)	ns (>0,9999)
**31**	ns (>0,9999)	ns (0,9964)	ns (0,9927)	ns (0,9972)	ns (0,9943)	ns (>0,9999)
**32**	ns (0,1816)	ns 0,9964)	ns (0,9927)	ns (0,6341)	ns (0,4285)	ns (>0,9999)
**33**	ns (0,0980)	ns (0,9964)	ns (0,9927)	ns (0,5078)	ns (0,2894)	ns (>0,9999)
**34**	ns (0,0980)	ns (0,9964)	ns (0,9927)	ns (0,5078)	ns (0,2894)	ns (>0,9999)
**35**	ns (0,3224)	ns (>0,9999)	ns (0,9763)	ns (0,6341)	ns (0,2134)	ns (0,9899)
**36**	ns (0,6872)	ns (0,9964)	ns (0,9019)	ns (0,7548)	ns (0,3327)	ns (0,9899)
**37**	ns (0,5473)	ns (0,9148)	ns (0,6032)	ns (0,3875)	ns (0,0700)	ns (0,9899)
**38**	ns (0,2318)	ns (0,9148)	ns (0,6032)	ns (0,1956)	* (0,0172)	ns (0,9899)
**39**	ns (0,2318)	ns (0,9148)	ns (0,6032)	ns (0,1956)	* (0,0172)	ns (0,9899)
**40**	ns (0,3741)	ns (0,9148)	ns (0,6032)	ns (0,2819)	* (0,0360)	ns (0,9899)
**41**	ns (0,3922)	ns (0,4248)	ns (0,4304)	# (0,0415)	* (0,0172)	ns (0,9899)
**42**	ns (0,1216)	ns (0,6976)	ns (0,9466)	# (0,0415)	ns (0,0565)	ns (0,9274)
**43**	ns (0,5876)	ns (0,3039)	ns (0,2799)	# (0,0415)	* (0,0172)	ns (0,9899)
**44**	ns (0,7799)	ns (0,2053)	ns (0,3511)	# (0,0415)	ns (0,0565)	ns (0,9274)
**45**	ns (0,5876)	ns (0,3039)	ns (0,2799)	# (0,0415)	* (0,0172)	ns (0,9899)
**46**	ns (0,3922)	ns (0,4248)	ns (0,4304)	# (0,0415)	* (0,0172)	ns (0,9899)

**47**	ns (0,3922)	ns (0,4248)	ns (0,4304)	# (0,0415)	* (0,0172)	ns (0,9899)
**48**	ns (0,7799)	ns (0,2053)	ns (0,1662)	# (0,0415)	* (0,0172)	ns (0,9899)
**49**	ns (0,7799)	ns (0,2053)	ns (0,3511)	# (0,0415)	ns (0,0565)	ns (0,9274)
**50**	ns (0,7799)	ns (0,2053)	ns (0,3511)	# (0,0415)	ns (0,0565)	ns (0,9274)
