# Supplementary figures and images for: An increase in Semaphorin 3A biases the axonal direction and induces an aberrant dendritic arborization in an in vitro model of human neural progenitor differentiation
Source: Cell Biosci. 2022 Nov 8;12:182. doi: 10.1186/s13578-022-00916-1 (PMC9641809; doi:10.1186/s13578-022-00916-1)

**A**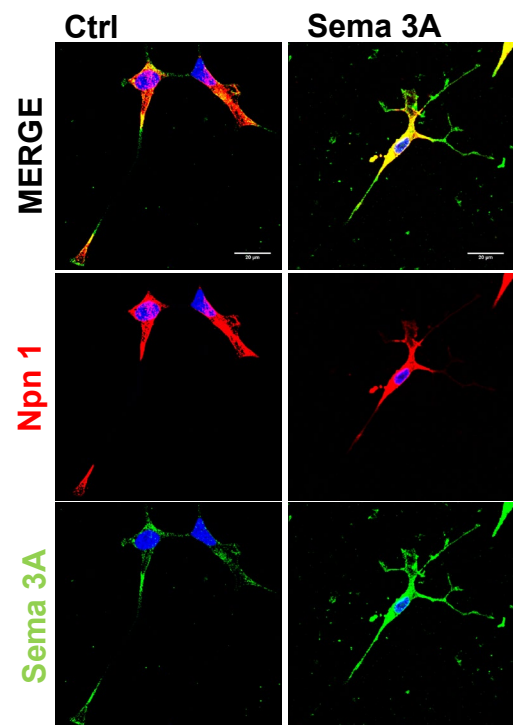**B**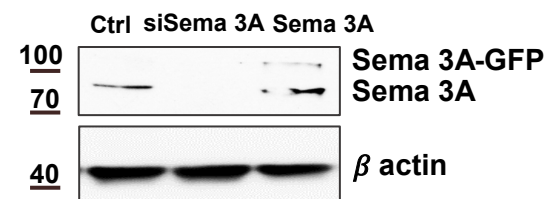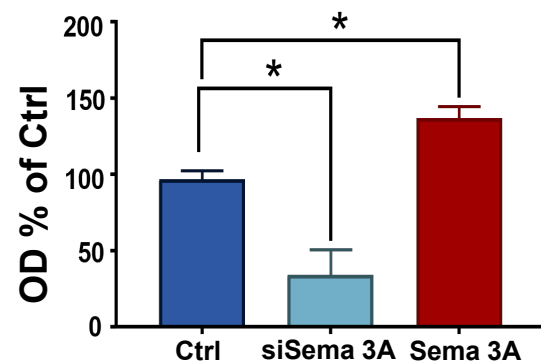**C**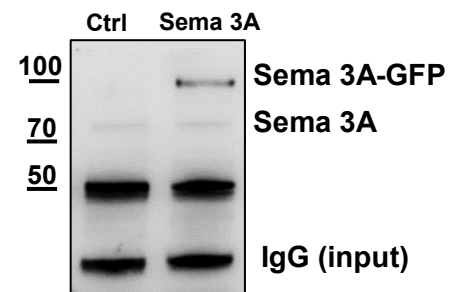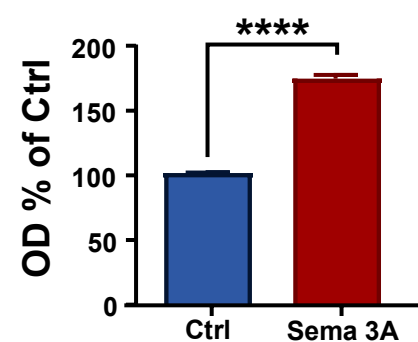**D**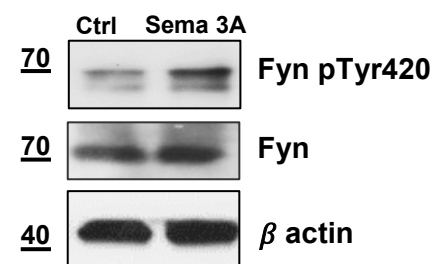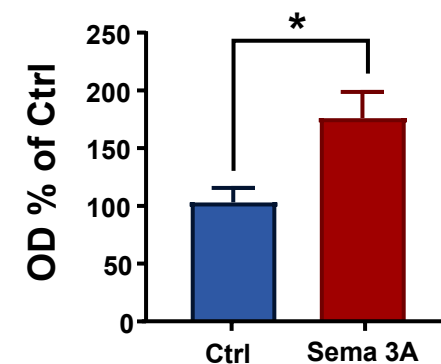**E**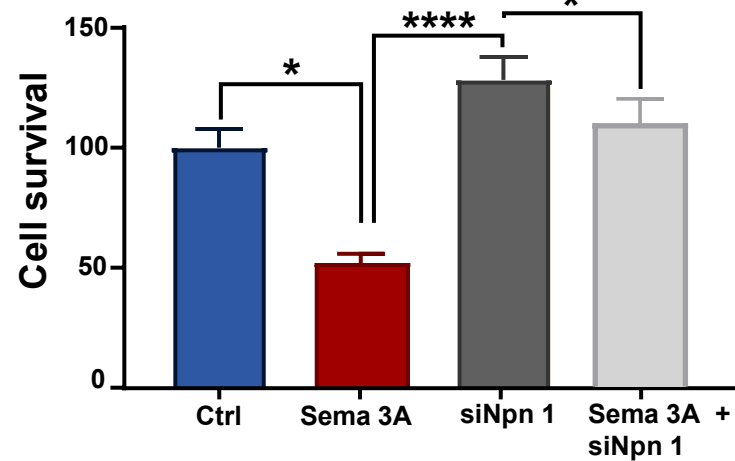**F**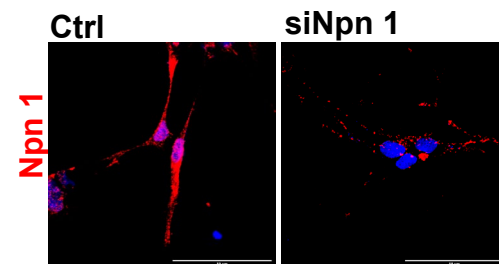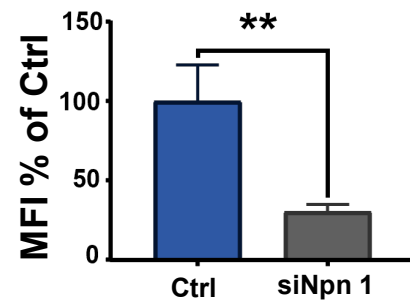**G**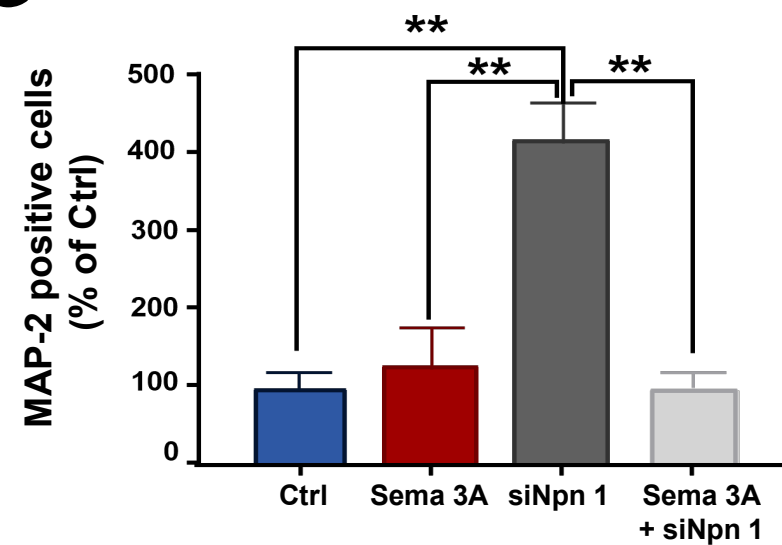**Fig.S1**

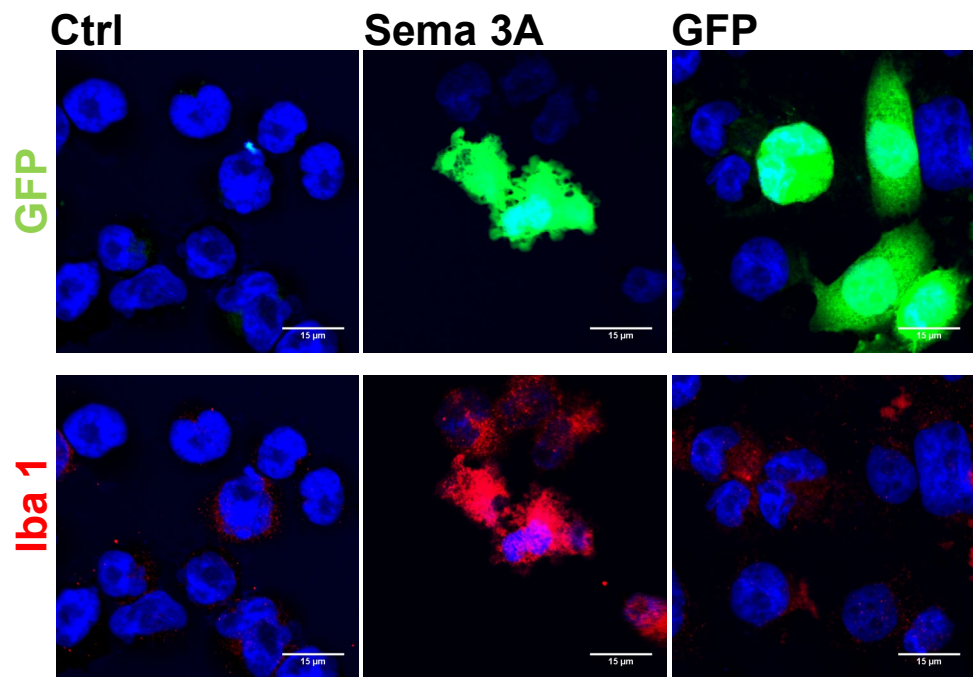

**Fig.S2**

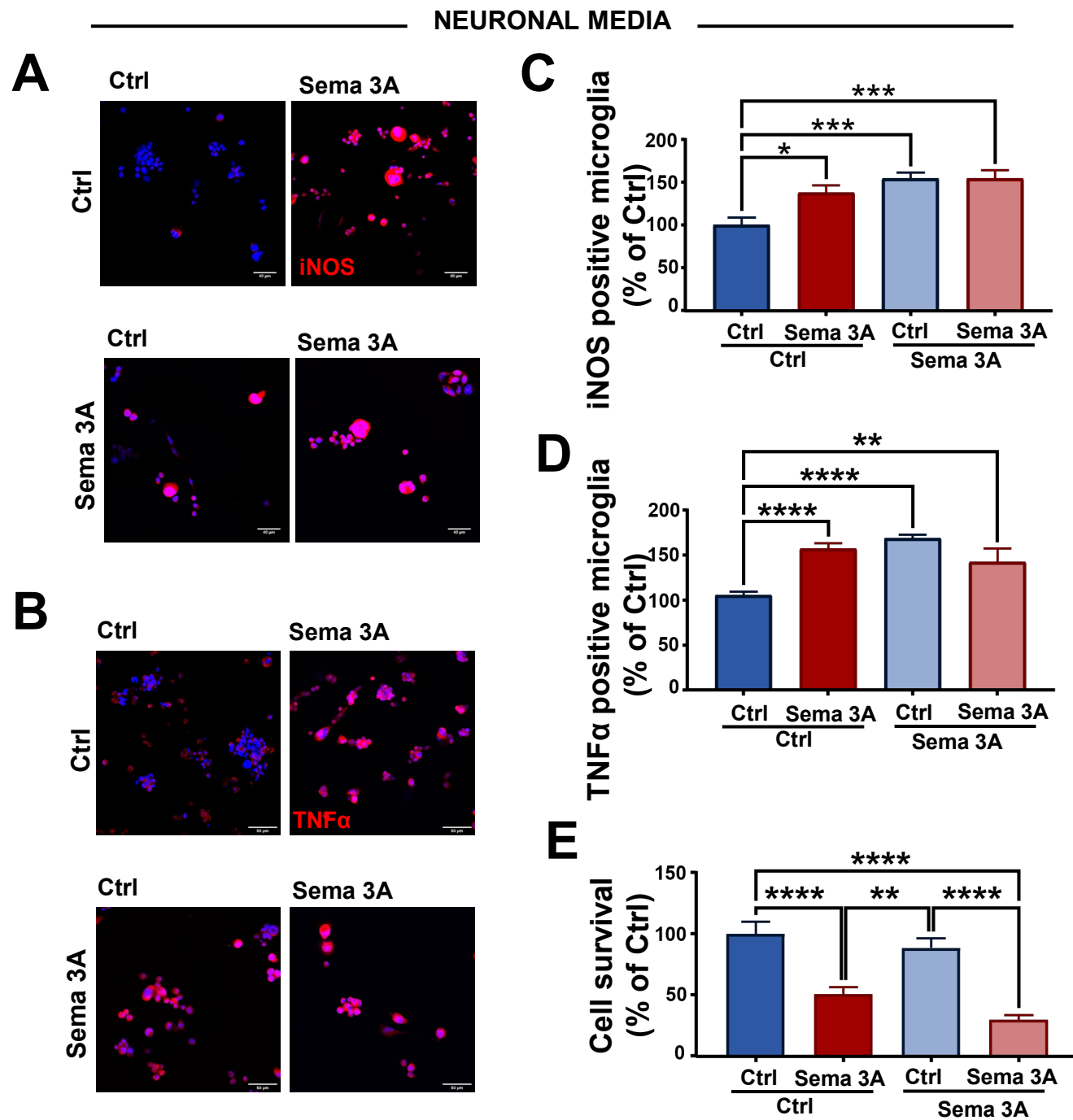

**Fig.S3**

Supplement: Supplementary file 2 — Additional file 2: Fig.S1. Characterization of Ctrl, Sema 3A-GFP, siSema 3A, siNpn 1 and Sema 3A+siNpn 1 neurons 48 h after transfection. (A) IF analysis with Sema 3A (green) and Npn 1 (red) of neurons transfected (Sema 3A) or not (Ctrl) with Sema 3A-GFP. Scale bar: 20μm. Figures were acquired by 40x objective and cropped in order to visualize single neuron and highlight the preferential Sema 3A localization on the apical dendrites in Sema 3A transfected neurons. (B) WB analysis of neurons overexpressing Sema 3A-GFP (Sema 3A) or in which Sema 3A is silenced (siSema 3A) 48 h after transfection. Non-transfected neurons were used as control. Optical density (OD) analysis is reported below. Data are the mean ± SEM of three independent experiments and are expressed as % of Ctrl. One-way ANOVA followed by Tukey’s test for multiple comparisons. *P<0.05 vs Ctrl. (C) Representative WB analysis for Sema 3A of media from NP overexpressing Sema 3A-GFP (Sema 3A) or non-transfected Ctrl. OD analysis is reported below. Sema 3A levels were normalized for the corresponding IgG value (input). One-way ANOVA followed by Tukey’s test for multiple comparisons. N=3 ****P < 0.0001. (D) Representative WB analysis of Fyn pTyr420, Fyn and the correspondent β actin. OD analysis is reported below. Fyn pTyr420 levels were calculated as a ratio of Fyn pTyr420 relative to the corresponding Fyn OD values normalized to β actin (Fyn pTyr420/Fyn). N= 3. One-way ANOVA followed by Tukey’s test for multiple comparisons. *P < 0.05. (E) Extent of neuronal survival obtained by counting the number of DAPI positive nuclei before and after Sema 3A transfection as well as in Npn 1 silencing and Ctrl (non-transfected neurons). Data are the mean ± SEM of three independent experiments in triplicate. One-way ANOVA followed by Tukey’s test for multiple comparisons. *P<0.5, ****P<0.0001. (F) Staining quantification of Npn 1 RNA silencing (siNpn 1) expressed as mean fluorescence intensity (MFI). MFI was normalized on [file 13578_2022_916_MOESM2_ESM.pdf]
